# Telomere-based risk models for the early diagnosis of clinically significant prostate cancer

**DOI:** 10.1038/s41391-020-0232-4

**Published:** 2020-05-04

**Authors:** Juan Manuel Rubio Galisteo, Luis Fernández, Enrique Gómez Gómez, Nuria de Pedro, Roque Cano Castiñeira, Ana Blanca Pedregosa, Ipek Guler, Julia Carrasco Valiente, Laura Esteban, Sheila González, Nila Castelló, Lissette Otero, Jorge García, Enrique Segovia, María José Requena Tapia, Pilar Najarro

**Affiliations:** 1 Department of Urology, Reina Sofía University Hospital, IMIBIC, UCO, Córdoba, Spain; 2 Life Length SL, Madrid, Spain; 3 Department of Urology, Infanta Margarita Hospital, Córdoba, Spain; 4 Instituto Maimónides de Investigación Biomédica de Córdoba (IMIBIC), Córdoba, Spain; 5 Sermes CRO, Madrid, Spain

**Keywords:** Diagnostic markers, Predictive markers, Cancer screening, Cancer screening, Diagnostic markers

## Abstract

**Background:**

The objective of this study was to explore telomere-associated variables (TAV) as complementary biomarkers in the early diagnosis of prostate cancer (PCa), analyzing their application in risk models for significant PCa (Gleason score > 6).

**Methods:**

As part of a larger prospective longitudinal study of patients with suspicion of PCa undergoing prostate biopsy according to clinical practice, a subgroup of patients (*n* = 401) with PSA 3–10 ng/ml and no prior biopsies was used to evaluate the contribution of TAV to discern non-significant PCa from significant PCa. The cohort was randomly split for training (2/3) and validation (1/3) of the models. High-throughput quantitative fluorescence in-situ hybridization was used to evaluate TAV in peripheral blood mononucleated cells. Models were generated following principal component analysis and random forest and their utility as risk predictors was evaluated by analyzing their predictive capacity and accuracy, summarized by ROC curves, and their clinical benefit with decision curves analysis.

**Results:**

The median age of the patients was 63 years, with a median PSA of 5 ng/ml and a percentage of PCa diagnosis of 40.6% and significant PCa of 19.2%. Two TAV-based risk models were selected (TAV models 1 and 2) with an AUC ≥ 0.83 in the full study cohort, and AUC > 0.76 in the internal validation cohort. Both models showed an improvement in decision capacity when compared to the application of the PCPT-RC in the low-risk probabilities range. In the validation cohort, with TAV models 1 and 2, 33% /48% of biopsies would have been avoided losing 0/10.3% of significant PCa, respectively. The models were also tested and validated on an independent, retrospective, non contemporary cohort.

**Conclusions:**

Telomere analysis through TAV should be considered as a new risk-score biomarker with potential to increase the prediction capacity of significant PCa in patients with PSA between 3–10 ng/ml.

## Introduction

Prostate cancer (PCa) is a major cause of morbidity and mortality worldwide with 450,000 new cases of PCa in 2018 in the EU, representing 10.6% of all cancers [1]. Current screening in males at risk consist of measurement of the Prostate Specific Antigen testing (PSA) levels. If PSA is ≥3 ng/ml, then a biopsy should be considered [2]. Although the levels of PSA are routinely used in the decision to perform a prostate biopsy, this diagnostic procedure is associated with low specificity, biopsy complications, and overdiagnosis [3, 4]. Overdiagnosis and overtreatment of indolent disease could be a major issue regarding cost and management of treatment complications [5, 6].

The development of new prediction models based on multiple biomarkers for PCa that combine PSA screening with other assays and/or imaging techniques is evolving rapidly [7]. Telomeres have emerged in recent years as potential biomarkers of risk in a variety of malignancies [8–12]. In the case of PCa, previous studies showed non-conclusive data about the association of telomere biology with the diagnosis of PCa [8, 13–15], but also suggested an association with PCa prognosis [16, 17]. Overall, these studies suggest that peripheral blood mononucleated cell (PBMC) telomeres might be used as a biomarker in PCa.

High-throughput quantitative fluorescent in situ hybridization (HT Q-FISH) allows for highly accurate and sensitive measurements of telomere length (TL) and percentage of short telomeres in large human sample sets [18]. Our group has recently extended HT Q-FISH capabilities by adding image capture and processing and software systems into an integrated Telomere Analysis Technology (TAT^®^), including the full validation of TAT [19]. This methodology and associated software allow for the evaluation of a large number of telomere-associated variables (TAV) in the cell population, greatly increasing the information derived from each sample analyzed. The TAV describe the full distribution of telomere lengths, the proportions of short and long telomeres, or the percent of cells with a specific telomere length average, among other parameters, generating a ‘TAV signature’ that can be used in comparative studies.

The main objective of this study was to determine if the parameters associated with the biology of telomeres are valid as biomarkers in PCa through a prospective evaluation of the TAV-based models on their ability to predict positive biopsies for significant PCa (Sig PCa).

## Methods

This prospective, bicentric, study was carried out at the Urology Department of two hospitals from Southern Spain. The study was not associated to any specific therapy or drug. The protocol was approved by the local institutional review board and health authorities (Reina Sofía University Hospital). All subjects provided signed consent for participation in the study.

### Population

Patients were prospectively recruited. Eligible patients were >18 years of age, classified as PCa-risk patients with PSA > 3 ng/mL and/or suspicious digital rectal examination (DRE), and that underwent a prostatic needle biopsy. From the global cohort a subset of patients was selected that met additional requirements; no prior biopsies, PSA between 3 and 10 ng/mL, and no prior use of alpha-5-reductase inhibitors. Patients were excluded if they had any active liver, lung or kidney disease, or severe infection; mental health disability preventing the signing of the informed consent forms or ability to follow procedures; increased risks during blood draws; or other diagnosed tumors.

All patients underwent transrectal ultrasound-guided biopsy with at least 12 cores. Specimens were evaluated by three uropathologists following the recommendations of the International Society of Urological Pathology (ISUP) [20].

### Telomere analysis and Telomere-Associated Variables (TAV)

The study of telomeres from the clinical samples was performed in the laboratories of Life Length SL (Madrid, Spain) within the scope of CLIA (99D2112462) and ISO 15189 quality standards. TAV are determined by TAT and by quantitative Telomeric Repeat Amplification Protocol (Q-TRAP). A summary list of the TAV is presented in Supplementary Table 1. The TAV can generate descriptive statistics of telomere length, values for each telomere length percentile, percentages of telomeric length values (ShortTel), percentages of cells with specific telomere values (ShortCell), and parameters of dispersion. The combination of these values can generate unique profiles, or TAV signatures, that can be used to define patterns of diagnostic and prognostic use.

TAT is based on HT Q-FISH [18] and its validation at Life Length was described in detail elsewhere [19]. Briefly, 10 ml of venous blood is collected in K2-EDTA tubes and PBMC are isolated by standard ficol-hypaque gradient centrifugation. The leucocyte single-cell suspension is then analyzed by HT-Q-FISH to detect and quantify individual telomere length. A fluorescent Peptide Nucleic Acid probe (PNA) that recognizes telomere repeats (sequence: Alexa488-OO-CCCTAACCCTAACCCTAA, Panagene) is used for hybridization. The test is performed in 384 well plates and its scalability allow its application in real life. The images of the nuclei and telomeres are captured by a high-content screen system. The intensities of fluorescence are translated to base pairs through a standard regression curve which is generated using control cell lines with known TL. Quantitative image acquisition is performed on a High Content Screening Opera System (Perkin Elmer) using a 40 × 0.95NA water immersion objective. Data were analyzed using proprietary software to generate all TAV validated through the same CLIA and ISO15189 laboratory standards.

### Model generation and validation

**Fig. 1:**
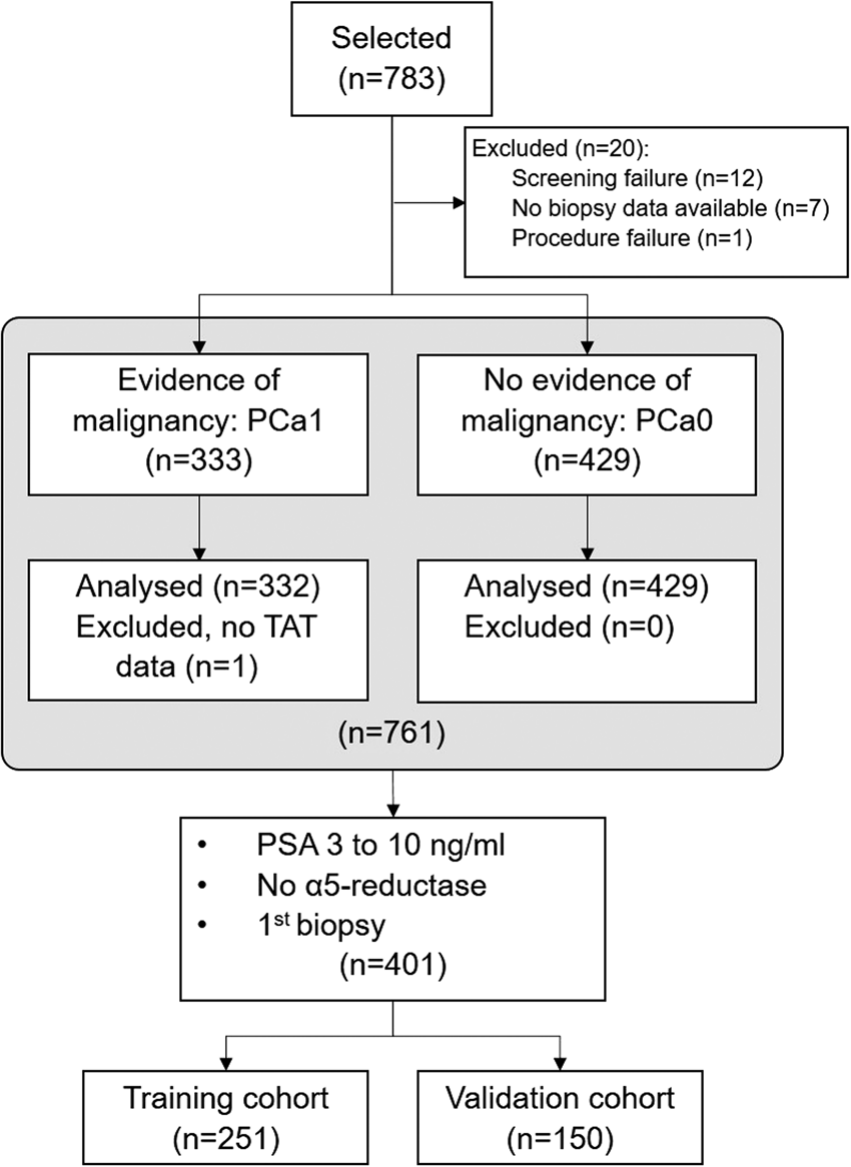
Patient selection for this study. Process of patient selection from the global cohort of the Oncocheck study (*n* = 783) to the cohort used in this study for risk model generation and validation (*n* = 401).

Data harmonization began with the global cohort with clinical data available for 783 samples (Fig. 1). The clinical data from the cohort were then joined with the available data from telomeric analysis (see below), using the sample code as the joining key. Clinical data used were presence or absence of cancer diagnosis, age, PSA, free PSA, DRE results along with TAV and presence of Sig PCa (Gleason score > 6) were used to generate the models. Of the 761 samples with both clinical and telomeric data available, the selection of samples following aforementioned criteria left 401 samples for model generation.

Of the 401 samples, 251 were used for model training (training cohort), and 150 for model validation (validation cohort). For each considered combination of TAV and clinical variables, 3,500 models were generated. For each model, 75% of the samples in the training set were randomly selected to train the model, and the remaining 25% used to evaluate the model.

For model generation, all numeric variables were normalized using Z-score. The dataset was then transformed using principal component analysis (PCA). The software KNIME (Zürich, Switzerland) was used for data processing. Random Forest was used to generate the models, and their probability thresholds evaluated at 0.1 intervals. Performance of each combination of model and threshold was determined firstly by the number of cases of Sig PCa that would not have been sent to biopsy (lower equals better performance). Secondly, for those selected that had an equal number cases of Sig PCa that would not have been sent to biopsy, by the number of samples without cancer or with non-significant cancer that would not have been sent to biopsy (higher equals better performance).

Receiver Operating Characteristic (ROC) curves were used to characterize the performance of the models and allowed for comparison with the performance offered by other options. The Prostate Cancer Prevention Trial (PCPT) risk calculator 2.0 + free PSA (http://riskcalc.org/PCPTRC/) was used as a standard to compare the models [21], and logistic regression models were also generated for certain high-interest clinical variables, such as PSA. The decision curves were also used for the model comparisons which represent the net benefit of the models according to different thresholds used for clinical decision [22]. All the statistical analyses were performed using R V.3.2.3 (R Foundation for Statistical Computing, Vienna, Austria; https://www.R-project.org/) and KNIME [23].

### Retrospective evaluation of the models

We performed an extensive evaluation of the selected generated models in an independent cohort: a set generated from patient samples collected from the ONCOVER study cohort [24]. This cohort generated a biobank of cryopreserved blood samples from patients at risk of PCa who underwent transrectal prostate biopsy according to clinical practice. Briefly, patients with PSA levels of 3–10 ng/mL and age 55–80 years were selected within the cohort for this study. Biopsy specimens were analyzed by expert urologic pathologists according to the ISUP 2005 modified criteria [25].

This retrospective cohort was selected according to PBMCs availability and following codification criteria that allowed a distribution of the samples as similar as possible to the prospective cohort to avoid discrepancies based on unrepresented classes, age or clinical variable distribution. In order to perform this balancing, PSA and age, which were originally continuous variables, were discretized through a binning process. For the binning process, a total of three bins for both age and PSA were selected in the prospective validation set, and the same binning was applied to the entire retrospective set. Subsequently, a binary code was formed to uniquely identify each possible combination of age bin, PSA bin, cancer diagnosis, and presence of Sig PCa. Two bits were used for the age classification, two bits for the PSA classification, one bit for cancer diagnosis, and one bit for the presence of Sig PCa. A total of 83 balanced samples were used in the evaluation.

## Results

A total of 401 samples of patients representing the intended use population were selected for model training (*n* = 251) and validation (*n* = 150), and their characteristics are shown in Table 1. The median age of the patients was 63 years (IQR, 43–86) and the median PSA was 5 ng/mL. In this population 77 patients (19.2%) were diagnosed with Sig PCa and 324 (80.8%) with non-significant or free of PCa. Significant risk factors included the levels of free PSA (*p* = 0.001) and a suspicious DRE (*p* = 0.008). Sig PCa was diagnosed in 48 (19.1%) of the patients with samples used for model training, and in 29 (19.3%) of the patients with samples used for validation. The training and validation cohorts were not statistically different (Supplementary Tables 2, 3).

**Table 1:** Characteristics of the patients included in the study.

Variable	Global (*n* = 401)	Significant PCa (*n* = 77)	Non-significant or no PCa (*n* = 324)	*p* value
Age, years, median (IQR)	63 (58–69)	67 (61.7–71.5)	62 (57–68)	≤0.01
Family background, *n* (%)	71 (17.7)	18 (23.4)	53 (16.4)	0.15
BMI, Kg/m^2^, median (IQR)	27.7 (25.3–30.4)	27.5 (5.3)	27.7 (25.4–30.4)	0.41
PSA, ng/mL, median (IQR)	5.0 (4.1–6.4)	5.4 (4.1–6.5)	5.0 (4.1–6.4)	0.24
Free PSA, ng/mL, median (IQR)	18 (14–25)	16 (11–21.5)	19 (15–25)	≤0.01
Suspicious DRE, *n* (%)	62 (15.5)	19 (24.6)	43 (13.3)	≤0.01

*BMI* body-mass index, *DRE* digital rectal examination, *IQR* interquartile range, *PCa* prostate cancer, *PSA* prostate specific antigen.

### Model construction and validation

**Fig. 2:**
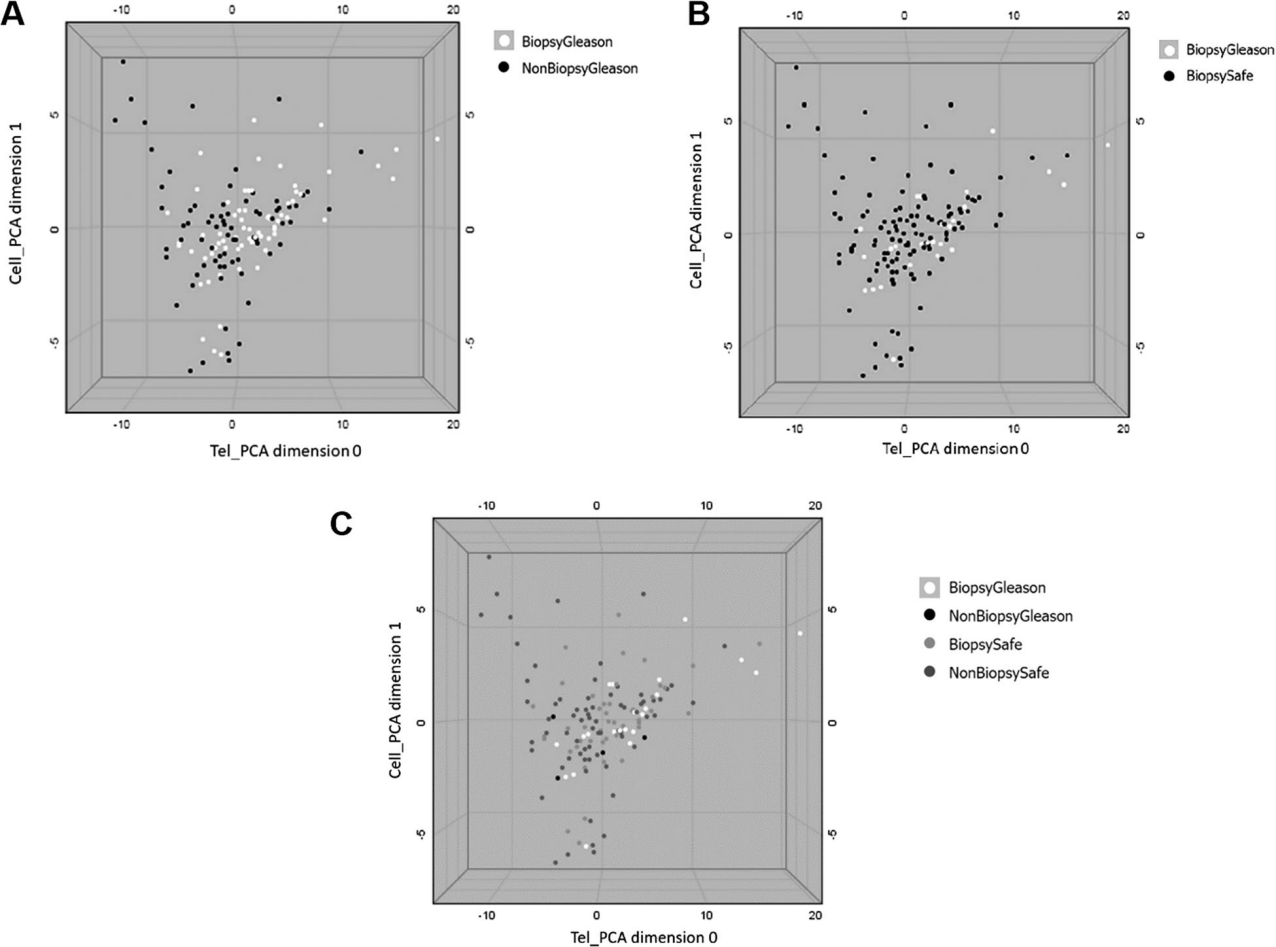
Principal component analysis using TAV representation with two PCA dimensions (*x* and *y* axis) against the absolute median deviation of the telomere intensities (MADI2) (*z* axis). **a** shows all patients included in the PCA using TAV as white dots representing patients that need to have a confirmatory biopsy (Biopsy Gleason) and black dots representing patients that do not require a confirmatory biopsy (No Biopsy Gleason). **b** shows all patients included in the PCA using TAV labeled according to their biopsy result as well as the significance or not of the diagnosed cancer. Black dots represent patients with no significant cancer according to their biopsy results (Biopsy safe) and white dots represent patients with significant cancer diagnosed after biopsy (Biopsy Gleason). **c** shows all patients included in the PCA using TAV labeled according to the prediction of the model. White dots represent patients with significant cancer found after biopsy that the model based on TAV indicates they should have a biopsy (Biopsy Gleason) that represent true positives, light gray dots represent patients that the model found should have a biopsy yet it was not necessary (Biopsy safe) that represent false positives, dark gray dots represent patients that the model indicates should not have a biopsy and indeed they did not needed (Non biopsy safe) representing true negatives and black dots denote patients with cancer diagnosed after biopsy that the model indicated should not have a biopsy (Non biopsy Gleason) that represent false negatives.

All patient samples were processed to obtain a TAV signature from PBMCs’ telomeres. After model generation, a detailed analysis was manually performed to look for maximal benefit (% of saved biopsies) with minimal impact (no false negatives). An example of the outcome of the PCA analysis is shown in Fig. 2.

**Fig. 3:**
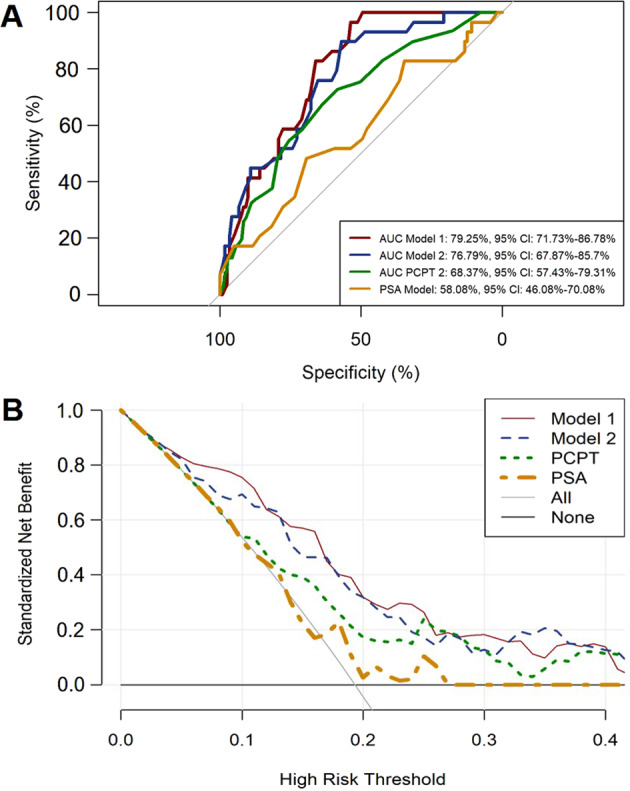
Performance of the TAV-based models. Area under receiver operating characteristic (AUC) curves are shown to compare performances of the two TAV-based models in the validation cohort with the PCPT-RC and PSA (**a**). The corresponding net benefit analysis for the two models and comparisons are shown in (**b**).

The best models were selected following the described methodology. These were named model 1 and model 2. The performance of the TAV signature to improve in decision capacity was evaluated by the area under ROC curves (AUC) as shown in Fig. 3. The AUC of the two selected validation models, 1 and 2, were 79.25 and 76.79%, respectively; both were higher than the AUC of the PCPT-RC, 68.37%, and PSA, 58.08% (Fig. 3a).

Regarding the net benefit calculated for both models and when compared to the standardized net benefit based on PCPT-RC or PSA, model 1 and model 2 showed an improvement compared to the application of the PCPT-RC and PSA, in the low-risk probabilities range (Fig. 3b).

Within the validation cohort, model 1 demonstrated a positive predictive value (PPV) of 0.29 and a negative predictive value (NPV) of 1.0 for prediction of Sig PCa and model 2 a PPV of 0.33, and a NPV of 0.95 (Table 2), meaning that with model 1, 33% of biopsies would be avoided without losing any cases of Sig PCa. With model 2, 48% of biopsies would be avoided at the cost of losing 10.3% cases of Sig PCa.

**Table 2:** Performance of the TAV models in the validation cohort.

TAV model 1				
	Biopsy result			
	Significant PCa	Non-significant PCa	Total	*Sensitivity: 1.00 (95%CI, 1.00–1.00)*
High risk	29	71	100	*Specificity: 0.51 (95% CI, 0.41–0.61)*
Low risk	0	50	50	*PPV: 0.52 (95% CI, 0.42–0.62)*
Total	29	121	150	*NPV: 1.00 (95% CI, 1.00–1.00)*
TAV model 2				
	Biopsy result			
	Significant PCa	Non-significant PCa	Total	*Sensitivity: 0.94 (95%CI, 0.87–1.00)*
High risk	26	52	78	*Specificity: 0.69 (95% CI, 0.60–0.78)*
Low risk	3	69	72	*PPV: 0.60 (95% CI, 0.49–0.71)*
Total	29	121	150	*NPV: 0.96 (95% CI, 0.91–1.00)*

### Retrospective validation

A validation set with 83 samples was generated from a retrospective cohort. The models 1 and 2 were applied for performance assessment on the retrospective validation cohort. The AUC was 0.77 and 0.78, respectively (data not shown).

Within the retrospective validation, model 1 demonstrated a PPV of 0.24 (95% CI: 0.12–0.38) and the NPV of 0.98 (95% CI: 0.87–1.0) and model 2 showed a PPV of 0.29 (95% CI: 0.15–0.45) and a NPV of 0.98 (95% CI: 0.93–1.0) to predict Sig PCa (Supplementary Table 4). According to this validation, 39% of the cohort would have been avoided undergoing biopsies at a cost of missing 7.6% of cases with Sig PCa.

## Discussion

Here we have described the use of TAV in the generation of predictive models of Sig PCa which could help during early diagnosis to reduce the number of unnecessary biopsies. We showed that TAV-based models have better performance (AUC > 76%) than the PCPT-RC (AUC 69.36%) for predicting Sig PCa in low-risk PCa patients undergoing their first biopsy. By using TAV-based models, 33 to 48% of the biopsies could have been avoided, at a cost of not detecting 0–10% of Sig PCa. Our preliminary results suggest that TAV-based models could improve the predictive power of the current initial diagnostic pathways.

Most patients undergoing PSA screening are referred for a biopsy when the levels are ≥3 ng/mL [26]. This pathway, associated with false-positive results, leads to more than 2 million biopsies performed each year in the US and Europe, a no-painless technique which leads to complications [3, 4, 24, 27]. It would be highly desirable to develop new clinical tools for the assessment of PCa risk. Likewise, both overdiagnosis and, most importantly, over-treatment of clinically indolent or non-significant PCa can result in an increase in anxiety, social and functional problems, as well as an increase in morbidity and alterations in the quality of life.

Several types of molecular markers that could be useful for risk evaluation, diagnosis, and prognosis of PCa have been identified [28, 29]. Most of these tests, derived from serum and urine, are based on PSA-related measurements, determination of particular mRNAs levels and/or proteins or exosomal biomarkers. None of them have been truly implemented so far in standard clinical practice. This is probably due to various reasons including relatively low sensitivity [30] or the need of performing rectal stimulation before urine sample collection [29, 31], which it is a nuisance for the patient. Another concern is the high price of some of these tests.

Telomere shortening is associated with increased all-cause mortality risk in the general population [32]. In the case of PCa, a recent meta-analysis of the available evidence showed that PCa was negatively associated with short telomeres in PBMC (OR, 0.81; 95% CI: 0.73–0.91) [33]. Earlier studies have shown that short TL in PBMC were indicative of decreased PCa risk, but the results were not statistically significant [8, 13, 14]. Conversely, a case-control study found that longer TL in PBMCs was modestly associated with higher risk of PCa [15]. Additionally, recent studies have suggested that TL was also associated with worse PCa-related prognosis. A long-term study found that patients with longer PBMC telomeres have higher overall mortality in patients with PCa [16]. A second study showed that patients with long TL had a significantly worse PCa-specific and metastasis-free survival compared to patients with short TL [17]. Although the evidence so far is inconclusive, it is possible that current views are limited by the sensitivity of the techniques used, including the fact that conclusions are based on single telomere measurements (i.e., average TL in the sample).

Another limitation of some of the previous studies on PBMC TL measurements is the lack of accuracy and the inherent variability associated to the techniques used. For example, quantitative polymerase chain reaction (Q-PCR) is known to have intra-assay coefficients of variation as high 10% [34, 35]. Also, Q-PCR does not provide information about very short telomeres and comparability between studies is often problematic [36]. A high-throughput and more accurate tool such as the TAT used in this study, a Q-FISH-based platform, can obtain data on each telomere individually. This provides valuable information derived from the TL variations within a cellular sample and within each of the cells in it. TAT could help to reach more conclusive results on telomeres that can then be used to calculate PCa risk. The telomere-based assays used in this study has been conducted under ISO15189 accreditation, which is an absolute requirement for biomarker development and ensures the analytical performance of the method [19]. Direct comparison with other diagnostic markers under development is not possible in the context of the presented study, as paired data were not available and different cohorts were evaluated. However, our results in this study indicated an AUC higher than 0.76 that is within the range of 0.74–0.90 shown by other biomarkers such as SelectMdx, PHI, 4 K score, STHLM3 score [37–39], or proteomics panels, clearly reinforcing its putative value in PCa diagnoses pathway.

We have been able to assess TAV as biomarkers including data showing the effects of the intervention on clinical outcomes using a contextual analysis on the specific target population. However, further studies will be required to support the use proposed. We are aware of the limitation of the cohort as all patients were derived from a small geographical area and included no racial diversity. Furthermore, 12-core transrectal prostate biopsy is known to have limitations in comparison to trans perineal template biopsy or adding target biopsy to mpMRI lesions, techniques that could have increase the rate of Sig PCa detected and decreased the NPV of the marker [40, 41]. A multicentric, international, prospective study, including mpMRI, to evaluate the efficacy of the test is ongoing (ClinicalTrials.gov Identifier: NCT04124900). The use of TAV-based models could benefit the early diagnosis of Sig PCa in patients with elevated PSA levels.

## Supplementary information

Supplementary Materials
