# Supplementary material for: Telomere-based risk models for the early diagnosis of clinically significant prostate cancer
Source: Prostate Cancer Prostatic Dis. 2020 May 4;24(1):88–95. doi: 10.1038/s41391-020-0232-4 (PMC8012205; doi:10.1038/s41391-020-0232-4)
Supplement: Supplementary file 1 — Supplementary Materials [file 41391_2020_232_MOESM1_ESM.docx]

**SUPPLEMENTARY MATERIALS**

**for**

**Telomere-based risk models for the early diagnosis of prostate cancer**

Juan M. Rubio Galisteo *et al.*

**Supplementary Table 1.** Telomere-associated variables (TAV)

**Supplementary Table 2.** Characteristics of the patients included in the Discovery set compared to the full prospective cohort

**Supplementary Table 3.** Characteristics of the patients included in the Validation set compared to the full prospective cohort

**Supplementary Table 4.** Performance of the TAV models in the validation retrospective cohort

| **Supplementary Table 1.** Telomere-associated variables (TAV) | | | |
| --- | --- | --- | --- |
| ***Variable type*** | ***Variable name*** | ***Description*** | ***Number of output values*** |
| Descriptive statistics | Total Nuclei | Total number of nuclei in the sample. | 1 |
|  | StDevPromedioI2 | Standard deviation of the I2 median of the wells in a sample | 1 |
|  | CoVPromedioI2 | Variation Coefficient of the I2 median of the wells in a sample | 1 |
|  | LengthI2Normalized | TL corresponding to the normalization of the median of intensities | 1 |
|  | Length20Percentil | TL corresponding to the normalization of the 20th percentile of the intensities of a sample | 1 |
|  | TamPromedio | Average nucleus size per sample | 1 |
|  | MedianI2 | Median of intensities per sample | 1 |
|  | MedianI2Normalized | Normalization of the median of intensities per sample | 1 |
|  | LengthAVG_I2 | Average telomeric length of the telomeres in a sample | 1 |
|  | SDMedianLength | Standard deviation of the median telomere lengths of the wells of a sample. | 1 |
|  | CVMedianLength | Variation coefficient of the median TL of the wells of a sample | 1 |
| Telomere Length Percentiles | Percentil N | TL corresponding to the Nth percentile of a sample. Percentiles 1 and 100 are not included. | 98 |
| Percentages of telomeric length values (ShortTel) | ShortTel N | Percentage of telomeres of a sample with a TL under the threshold N | 80 |
| Percentages of cells with specific telomere values (ShortCell) | ShortCell N | Percentage of cells of a sample whose telomeres average a length under the threshold N | 40 |
| Dispersion | MAD-I2 | Median Absolute Deviation of the intensities of a sample | 1 |
|  | P1-99-Kpbs | Difference between the 99th percentile of the TLs of a sample and the 1st percentile of the telomere lengths of that sample. | 1 |
|  | P75-25-Kpbs | Interquartile of the TLs of a sample. | 1 |
| **Total TAV** | | | **232** |

| **Supplementary Table 2.** Characteristics of the patients included in the Discovery set compared to the full prospective cohort | | | |
| --- | --- | --- | --- |
| Variable | TRAINING SET  (n=251) | GLOBAL  SET (n=401) | *p value* |
| Age, years, median (IQR) | 63 (58-69) | 63 (58-69) | 0.80 |
| Family background, n (%) | 47 (18.7) | 71 (17.7) | 0.74 |
| BMI, Kg/m^2^, median (IQR) | 27.1 (25.1-30.1) | 27.7 (25.3-30.4) | 0.40 |
| PSA, ng/mL, median (IQR) | 5.0 (4.0-6.2) | 5.0 (4.1-6.4) | 0.42 |
| Free PSA, ng/mL, median (IQR) | 18 (13-24) | 18 (14-25) | 0.46 |
| Suspicious DRE, n (%) | 32 (14.3) | 62 (15.5) | 0.70 |
| Significant PCa (%) | 19.1 | 19.2 |  |
| Abbreviations: BMI=body-mass index; DRE=digital rectal examination; IQR=interquartile range; PCa=prostate cancer; PSA=prostate specific antigen. | | | |

| **Supplementary Table 3.** Characteristics of the patients included in the Validation set compared to the full prospective cohort | | | |
| --- | --- | --- | --- |
| Variable | TRAINING SET  (n=150) | GLOBAL  SET (n=401) | *p value* |
| Age, years, median (IQR) | 63 (58-69) | 63 (58-69) | 0.73 |
| Family background, n (%) | 24(16.0) | 71 (17.7) | 0.93 |
| BMI, Kg/m^2^, median (IQR) | 28.1 (25.7-30.8) | 27.7 (25.3-30.4) | 0.26 |
| PSA, ng/mL, median (IQR) | 5.2 (4.1-6.7) | 5.0 (4.1-6.4) | 0.26 |
| Free PSA, ng/mL, median (IQR) | 19 (15-25) | 18 (14-25) | 0.31 |
| Suspicious DRE, n (%) | 26 (17.3) | 62 (15.5) | 0.59 |
| Significant PCa  (%) | 19.3 | 19.2 |  |
| Abbreviations: BMI=body-mass index; DRE=digital rectal examination; IQR=interquartile range; PCa=prostate cancer; PSA= prostate specific antigen. | | | |

| **Supplementary Table 4.** Performance of the TAV models in the validation retrospective cohort | | | | |
| --- | --- | --- | --- | --- |
| **TAV model 1** | |  |  |  |
|  | Biopsy result | |  |  |
|  | Significant PCa | Non-significant PCa | Total | *Sensitivity: 0.92 (95%CI, 0.64-1.00)* |
| High risk | 12 | 39 | 51 | *Specificity: 0.44 (95% CI, 0.33-0.56)* |
| Low risk | 1 | 31 | 32 | *PPV: 0.24 (95% CI, 0.12-0.38)* |
| Total | 13 | 70 | 83 | *NPV: 0.97 (95% CI, 0.84-1.00)* |
|  |  |  |  |  |
| **TAV model 2** | |  |  |  |
|  | Biopsy result | |  |  |
|  | Significant PCa | Non-significant PCa | Total | *Sensitivity: 0.92 (95%CI, 0.64-1.00)* |
| High risk | 12 | 30 | 42 | *Specificity: 0.57 (95% CI, 0.45-0.68)* |
| Low risk | 1 | 40 | 41 | *PPV: 0.29 (95% CI, 0.15-0.45)* |
| Total | 13 | 70 | 83 | *NPV: 0.98 (95% CI, 0.87-1.00)* |
